# Who is using the morning-after pill? Inequalities in emergency contraception use among ever partnered Nicaraguan women; findings from a national survey

**DOI:** 10.1186/s12939-014-0061-y

**Published:** 2014-09-10

**Authors:** Mariano Salazar, Ann Öhman

**Affiliations:** Department of Public Health and Clinical Medicine, Epidemiology and Global Health, Umeå University, SE 901 85 Umeå, Sweden; Umeå Center for Gender Studies, Challenging Gender Excellence, Umeå University, Umeå, Sweden

**Keywords:** Emergency contraception, Epidemiology, Inequalities, IPV, Nicaragua

## Abstract

**Introduction:**

Few studies have described the inequalities in hormonal emergency contraception (HEC) use in developing countries. Thus, the main aim of this manuscript is to study socio-demographic inequalities in HEC use among Nicaraguan women, and to study if inequalities in HEC use arise from exposure to different forms of intimate partner violence (IPV).

**Methods:**

Data from a national cross-sectional study conducted from 2006 to 2007 was used. This study included data from 8284 ever partnered, non-sterilized women. Separate multivariate logistic regressions with each form of IPV were conducted to study how different forms of IPV were associated with HEC. Women’s age, residency, education, socioeconomic status, parity, and current use of reversible contraception were included in the multivariate logistic regressions to obtain adjusted odds ratios showing inequalities in HEC use.

**Results:**

Six percent of the women had ever used HEC (95% CI 5.1-6.9). Multivariate analyses showed that urban residency, higher education, and higher socioeconomic status were significantly associated with higher odds of ever using HEC, and age was associated with decreased odds of HEC use. A key finding of this study is that after controlling for socio-demographic factors, the odds of using HEC were higher for those women ever exposed to emotional IPV (AOR 1.58, 95% CI 1.16-2.00), physical IPV (AOR 1.82, 95% CI 1.30-2.55), sexual IPV (AOR 1.63, 95% CI 1.06-2.52), and controlling behavior by partner (AOR 1.51 95% CI 1.13-2.00) than those not exposed.

**Conclusions:**

This study provides sound evidence supporting the hypothesis that there are inequalities in HEC use even in countries where inequalities in use to other forms of contraceptive technology has been reduced. HEC use among Nicaraguan women is strongly influenced by individual factors such as age, residency, educational level, socioeconomic status, and exposure to different forms of IPV. It is paramount that actions are taken to diminish these gaps.

## Introduction

Increasing women’s access to modern contraceptive methods has been a key step to promoting women’s universally recognized sexual and reproductive rights around the world; that is, the right to control their own fertility and the right to enjoy sexual relations without always being associated with reproduction [1].

Unequal gender orders that promote unfair gender relations among men and women [2] constitute important factors hindering women’s attempts to exercise their sexual and reproductive rights. Hegemonic masculinity patterns [2] that foster unequal power relations between men and women can interfere with women’s reproductive rights in several ways. For example, in spite of increased use of modern contraceptive use in developed as well as developing countries [3–5], studies have shown that many women still experience reproductive control by their partners [6,7], a trait which has consistently been associated with negative reproductive health outcomes such as unintended pregnancies, induced abortions, and miscarriages or stillbirths [8,9].

Emergency contraception (EC) is another tool available to women who want to prevent unintended pregnancies and to maintain control of their fertility. The World Health Organization (WHO) defines EC as “*methods of contraception that can be used to prevent pregnancy in the first few days after intercourse*” [10]. Emergency contraception methods can be classified in two categories: emergency contraceptive pills containing hormones, and intrauterine devices. This manuscript addresses only hormonal emergency contraception use.

Hormonal emergency contraception (HEC) is recommended to women who have been exposed to sexual violence or to those whose regular contraceptive method failed or was not taken as prescribed [10,11]. Hormonal emergency contraception has been shown to be effective, safe and well accepted by women [12]. For example, a WHO randomized multi-country study including 4136 women showed that different regimes of HEC are effective in reducing unintended pregnancies [13]. In addition, data has shown that HEC use does not increase women’s risk of acquiring sexually transmitted infections or having an unwanted pregnancy [14,15].

Hormonal emergency contraception use varies significantly depending on the setting under study, the socio-demographic characteristics of the sample, and the country’s regulations regarding HEC use. For example, population-based studies have found that ever use of HEC varies between countries ranging from 1.4% in India [16], to 11% in Kenya and the US [17,18]. Women’s education seems to influence HEC use. In Kenya, Nigeria, and the US women higher education have been linked to higher HEC use [17,18]. This have been corroborated with data collected among US college students finding that HEC use ranges from 17% to 49% [19,20] which is higher than previously reported usage from demographic and health surveys in this setting (11%) [18]. Hormonal emergency contraception also varies according to women’s marital status and age. Never married women and cohabitating women use more HEC than those currently married [17,18], as do young women compared to older women [17,18]. Unprotected sex has also been linked to higher HEC use among certain population, such as Ethiopian college students [21]. Changes in legal regulations regarding access to HEC can increase its use. In the US, HEC has been available as an over-the-counter drug since 2006 [19]. Since becoming more available, ever use of HEC in the US has increased from 4% in 2002, to 11% in 2006–2010 [18].

### The Nicaraguan setting

Nicaraguan women’s use of modern contraception with methods such as pills, injectable contraceptives, intrauterine devices, condoms, and female sterilizations is common among both urban and rural women [22]. Although there is no data available on HEC use, HEC is available as an over-the-counter drug [23] and it is included in the national family planning guidelines [24]. Notwithstanding, women often obtain HEC from private vendors who can facilitate or hinder access to it. Ehrle and Sarker, in a study with Nicaraguan pharmacy personnel, found that most respondents had limited knowledge on HEC correct dosage and side effects [23]. In spite of their limited knowledge, seven out of ten respondents in Ehrle and Sarker’s study believed that HEC was useful in preventing unwanted pregnancies, and were willing to sell them to anyone [23].

Nicaraguan women face important reproductive challenges; unintended pregnancies are common, especially among young women [22]. Access to therapeutic abortions has been banned since 2007 [25], leaving few choices to women whose pregnancies might endanger their lives. Hormonal emergency contraception can contribute to decrease women’s risk of unwanted pregnancies and unsafe abortions; however, as described above, women’s socio-demographic characteristics can be important determinants of HEC use. Health inequality is defined as the “*differences, variations, and disparities in the health achievements of individuals and groups*” [26]. However, there are very few studies around the world assessing inequalities in HEC use, and none from Nicaragua. Thus, the main aim of this manuscript is to study socio-demographic inequalities in HEC use among Nicaraguan women. In addition, it has been highlighted that women exposed to intimate partner violence (IPV) are often exposed to reproductive coercion by their partners as well [8,9]. Nicaraguan women are no exception; they experience high IPV rates [22]. Therefore, we study if inequalities in HEC use arise from exposure to different forms of IPV.

## Methods

### Study design and sample selection

We used data from the 2006–2007 Nicaraguan Demography and Health Survey (NDHS), a national cross-sectional study [22]. The survey collected information at household (17209 households) and individual levels (14229 women aged 15–49 years) from women living in 142 municipalities across the country. It used a multi-stage cluster sampling based on information from the 2005 National Population and Household Survey. Detailed sampling information can be found elsewhere [22]. The response rate for the survey was 98% [22]. Our current study draws data from 8284 ever partnered, non-sterilized women who participated in the 2006–2007 NDHS. We have chosen this sample because in the NDHS, questions assessing HEC use were posted only to women who were not sterilized. We further limited our sample to ever partnered women as we wanted to assess if lifetime exposure to IPV was associated with ever HEC use.

### Measurements

The outcome variable HEC use was assessed by first asking the woman if she had ever heard of the morning-after pill. Interviewers could also tell the woman the concept of hormonal emergency contraception by using the following phrase, “Emergency contraception. A woman can take the pill 72 hours after she had sex without protection.” If the respondent reported that she knew about the method, the interviewers proceeded to ask her if she had ever used it. In our study, those who reported that they did not know about HEC were coded as if they had not used it.

Intimate partner violence was measured using questions from the WHO Multi-country Study on Women’s Health and Domestic Violence [27]. The instrument measures lifetime and last year exposure to different forms of IPV (emotional, physical, sexual and controlling behavior by partner) by a current or former partner. In this paper, we only use lifetime exposure to IPV, as we believe it will be associated with ever HEC use. Yelling, humiliation, intimidation, and threats were considered emotional IPV. Pushes, slaps, punches, kicks, hair pulling, burns, or use of weapons were considered physical IPV. A woman was considered to have experienced sexual IPV if she reported ever being forced to have intercourse by a partner. Questions measuring controlling behavior by partner included assessing if a woman’s partner had ever: limited her contact with family, friends, or health care; insisting on knowing where she was at all times; getting upset if a woman talks to other men; or always suspecting a woman of infidelity. A woman was considered to have been exposed to controlling behavior if she answered yes to any of the six questions described above. The WHO instrument has been used and validated worldwide [27]. In our study, all sub-scales have shown good internal validity with Cronbach’s alpha values ranging from 0.81 (emotional IPV) to 0.89 (physical IPV).

Data at the individual level included women’s age (years), residency (urban/rural), education (no education, primary school, high school or colleague), parity (number of times a woman has given birth), socioeconomic status, and current use of any reversible contraceptive methods. A household assets index was used as a proxy to measure women’s socioeconomic status. The index included questions on household availability of several kinds of electronic equipment (TV, radio, computers, etc.), means of transportation (bicycles, cars, boats, horses, etc.), and access to communication technology (landlines or cell phones). The index was created using principal component analysis, and later divided into quintiles. Contraceptive methods can be permanent, also named irreversible, or not. Irreversible methods include female and male sterilization. Thus, in this paper, reversible contraception was defined as women using any form of contraceptive method apart from female/male sterilization and HEC at the time of the survey data collection.

### Analysis

Stata (Version 12; StataCorp, College Station, Texas) was used to analyze the data. Univariate and bivariate statistics were used to describe the data. Sampling weights were introduced in the analysis of the data to correct for the survey multi-stage cluster design. Sampling weights used in this study were calculated by multiplying the household’s probability selection by the women’s selection probability. Women’s selection probability was inversely proportional to the number of women of childbearing age in the home. Detailed information on the construction of the sampling weights used in this study can be found elsewhere [22].

The T-test , Chi-square test, Fisher exact test and 95% confidence intervals were used when appropriate. Bivariate analysis is presented using weighted percentages. Separate multivariate logistic regressions with each form of IPV were conducted to study how different forms of IPV were associated with HEC. All socio-demographic variables were included in the multivariate logistic regressions to obtain adjusted odds ratios showing inequalities in HEC use. All associations were considered significant if p < 0.05. Multiplicative interaction was assessed between ever exposure to IPV and the other variables included in the model.

### Ethical considerations

This manuscript used a secondary data source. The data was collected by the Nicaraguan government and is freely available through an internet site (http://ghdx.healthmetricsandevaluation.org/record/nicaragua-reproductive-health-survey-2006-2007). The study is in compliance with the Helsinki Declaration; the database available for analysis did not contain information (names, addresses or identification numbers) that could be used to identify the women included in the study. The NDHS 2006–2007 final report states that fieldworkers were extensively trained on how to collect sensitive data without expressing judgment and maintaining confidentiality [22]. Also, it states that data on women’s exposure to IPV was collected only if confidentiality requirements were meet; that is, the field worker had to be alone with the interviewee, and she must have had ensured an environment where no one could listen to the conversation [22].

## Results

### Women socio-demographic characteristics

Women participating in this study were young, with a mean (SE) age of 28 (0.13) years. Five out of ten women lived in an urban setting, four out of ten had high school education or higher, and four out of ten were classified as having a medium-high or high socioeconomic status (Table 1). The mean parity was two point four children per woman, and six out of ten women were using some form of reversible contraception (Table 1). Lifetime exposure to different forms of IPV was common, ranging from 11% for sexual IPV to 54% for controlling behavior by partner (Table 1).

**Table 1:** **Women’s socio-demographic characteristics and lifetime exposure to intimate partner violence stratified by ever use of hormonal emergency contraception (HEC)**

**Characteristic**	**HE. contraception No**	**HE. contraception Yes**	**All**
	**n = 7901**	**n = 383**	**n = 8284**
	% (95% CI)	% (95% CI)	% (95% CI)
Age. Mean (SE)*	28.5 (0.14)	25.8 (0.34)	28.3 (0.13)
Residency. Rural*	48.2 (44.1-52.4)	9.1 (6.3-13.0)	45.9 (41.0-50.0)
Education*			
No education	15.7 (14.2-17.0)	0.5 (0.0-1.5)	14.8 (13.4-16.1)
Primary school	42.8 (40.9-44.6)	10.8 (7.2-15.8)	40.8 (39.0-42.7)
High school education	31.7 (29.9-33.6)	48.7 (42.1-55.4)	32.8 (30.9-34.7)
College education	9.8 (8.6-11.0)	40.0 (33.6-46.4)	11.6 (10.2-13.0)
Parity. Mean (SE)*	2.49 (0.03)	1.28 (0.06)	2.41 (0.04)
Socioeconomic status*			
Low	25.4 (22.2-28.3)	2.2 (1.0-4.7)	24.0 (21.1-26.8)
Medium - low	21.4 (19.8-23.2)	4.5 (2.6-7.2)	20.4 (18.7-22.1)
Intermediate	20.0 (18.4-21.6)	13.0 (9.3-17.6)	19.6 (18.0-21.2)
Medium - high	18.5 (16.5-20.2)	29.5 (23.5-36.2)	19.1 (17.2-20.9)
High	14.7 (12.9-16.7)	50.8 (43.8-57.8)	16.9 (14.8-19.2)
Current use of reversible			
Contraception. Yes	57.7 (56.1-59.2)	60.4 (53.9-66.6)	57.8 (56.3-59.3)
Emotional IPV. Yes†	43.6 (42.0-45.2)	49.3 (43.1-55.5)	43.9 (42.4-45.5)
Physical IPV. Yes†	23.2 (22.0-24.1)	27.7 (21.9-34.2)	23.4 (22.3-24.6)
Sexual IPV. Yes†	10.9 (10.0-11.8)	12.4 (8.6-17.4)	11.0 (10.1-11.9)
Controlling behavior by partner. Yes†	53.7 (52.0-55.4)	57.2 (50.7-63.3)	54.0 (52.3-55.6)

*T-test or Chi2 test, p < 0.05. †Lifetime exposure to intimate partner violence. Weighted percentages and 95% CIs shown.

### Hormonal emergency contraception use and bivariate associated factors

Six percent of the women had ever used HEC (95% CI 5.1-6.9). Women who used HEC were younger, lived mainly in urban areas, had lower parity and had higher education and socioeconomic status than those who not using HEC (p < 0.05) (Table 1). No significant association was found between different forms of lifetime IPV and HEC use (p > 0.05) (Table 1). Hormonal emergency contraception use was higher among women who reported using condoms, calendar-rhythm method, or withdrawal, and lower among those using injectable contraceptives and lactational amenorrhea methods as a contraceptive method at the time of the data collection (p < 0.05) (Table 2).

**Table 2:** Type of reversible contraceptives use among women*

**Type**	**HE. contraception No**	**HE. contraception Yes**	**All**
	**n = 4849**	**n = 243**	**n = 5092**
	**% (95% CI)**	**% (95% CI)**	**% (95% CI)**
Oral contraceptive	27.5 (25.8-29.3)	23.4 (17.9-30.0)	27.3(25.6-29.0)
Intrauterine device	8.3 (7.1-9.7)	8.0 (4.7-13.2)	8.3 (7.1-9.6)
Condom†	8.6 (7.5-9.7)	20.1 (14.4-27.3)	9.3 (8.25-10.5)
Injectable contraceptive†	49.6 (47.6-51.7)	38.2 (31.2-45.8)	48.9 (47.3-50.9)
Lactational amenorrhea method†	2.4 (1.9-3.1)	0.0 (0.0-0.0)	2.3 (1.8-2.9)
Calendar-rhythm method†	3.6 (2.9-4.4)	11.0 (6.6-17.3)	4.0 (3.3-4.9)
Withdrawal†	3.1 (2.4-3.9)	8.7 (4.9-15.1)	3.4 (2.8-4.2)

A comparison between those who have, and those who have not used hormonal emergency contraception (HEC). Weighted percentages and 95% CIs shown.

*Any method apart from female/male sterilization and HEC. † Chi2 test or Fisher’s exact test, p < 0.05. Percentages do not add to 100% due to the fact that the items are not mutually exclusive.

### Multivariate analysis

Adjusted estimates for all multivariate logistic regressions showed inequalities in HEC use. In all models, as age increased, the odds of HEC use decreased (p < 0.05). Women who lived in urban settings had with higher odds of HEC use than those who lived in rural settings (p < 0.05). Women with primary school education, secondary school education, or collage education had higher odds of ever using HEC than those with no education (p < 0.05). In all models, women who were classified as having medium-high or high socioeconomic status had higher odds of ever using HEC than those with low socioeconomic status. A key finding of this study is that after controlling for socio-demographic factors, the odds of ever using HEC were higher for those women exposed to lifetime emotional IPV (AOR 1.58, 95% CI 1.16-2.00), physical IPV (AOR 1.82, 95% CI 1.30-2.55), sexual IPV (AOR 1.63, 95% CI 1.06-2.52), and controlling behavior by partner (AOR 1.51, 95% CI 1.13-2.00) than those not exposed (Table 3). No significant multiplicative interaction was found between lifetime exposures to IPV, and women’s age, residency, education, socioeconomic status, parity or current use of reversible contraceptive methods (p > 0.05).

**Table 3:** **Associations between women’s lifetime exposure to IPV, socio-demographic characteristics, and use of hormonal emergency contraception (HEC)**

**Exposure**	**Lifetime emotional IPV**	**Lifetime physical IPV**	**Lifetime sexual IPV**	**Controlling behavior by partner**
	AOR (95% CI)*	AOR (95% CI)*	AOR (95% CI)*	AOR (95% CI)*
IPV				
No	1.00	1.00	1.00	1.00
Yes	1.58 (1.16-2.00)	1.82 (1.30-2.55)	1.63 (1.06-2.52)	1.51 (1.13-2.00)
Age (years)	0.92 (0.90-0.94)	0.92 (0.90-0.94)	0.92 (0.90-0.94)	0.92 (0.90-0.95)
Residency				
Rural	1.00	1.00	1.00	1.00
Urban	1.98 (1.34-2.94)	1.96 (1.32-2.90)	2.02 (1.36-3.00)	1.96 (1.32-2.91)
Education				
No education	1.00	1.00	1.00	1.00
Primary school	3.40 (1.12-10.3)	3.42 (1.13-10.36)	3.43 (1.13-10.39)	3.41 (1.12-10.31)
High school education	8.67 (2.87-26.22)	8.80 (2.92-26.56)	8.68 (2.88-26.14)	8.98 (2.98-27.04)
College education	21.4 (6.90-66.6)	22.3 (7.23-69.17)	21.47 (6.94-66.42)	22.6 (7.31-10.12)
Parity (number of children)	0.94 (0.82-1.07)	0.93 (0.82-1.06)	0.95 (0.83-1.07)	0.95 (0.87-1.08)
Socioeconomic status				
Low	1.00	1.00	1.00	1.00
Medium - low	1.22 (0.47-3.20)	1.24 (0.40-3.27)	1.25 (0.48-3.26)	1.26 (0.48-3.26)
Intermediate	2.26 (0.93-5.51)	2.32 (0.96-4.64)	2.29 (0.94-5.57)	2.35 (0.97-5.67)
Medium - high	4.25 (1.71-10.57)	4.29 (1.72-10.65)	4.33 (1.73-10.78)	4.38 (1.77-10.83)
High	7.20 (2.95-15.59)	7.30 (2.95-17.82)	7.29 (2.97-17.87)	7.45 (3.07-18.07)
Current use of reversible contraception†				
No	1.00	1.00	1.00	1.00
Yes	1.02 (0.74-1.39)	1.01 (0.74-1.38)	0.91 (0.67-1.25)	1.02 (0.75-1.39)

Adjusted odds ratios and 95% CIs shown. n = 8284.

*Adjusted for all variables included in the table. †Any method apart from female/male sterilization and HEC.

## Discussion

Women effective exercise of their reproductive rights depends on their use of means to avoid pregnancy including post-coital contraceptive methods. Our population-based findings show how differences among women’s socio-demographic characteristics (age, residency, education, and socioeconomic status) influence their experience with reproductive technology by either enhancing or lowering women’s use of HEC. One key and novel finding of our study is that women lifetime exposure to different forms of IPV is associated with higher odds of having ever used HEC even after adjusting for women’s socio-demographic characteristics.

### Socio-demographic inequalities in HEC use

Ever use of HEC among this population was low (6%), and within the middle range of HEC use reported by other population-based studies in India (1.4%) [16] and the US (11%) [18]. This finding might be explained by differences in the socioeconomic status of the populations under study. For example, in the Indian study, almost all women (92.7%) were classified as having a low socioeconomic status whereas only 24% of the Nicaraguan women in our study were classified as such. Although, Daniels et al. [18] do not report the socioeconomic status of the women included in their analysis, it is logic to think that women in a high-income country such as the US, have in average higher economic resources than women in low-income countries, and thus they are able to use more HEC than their poorer counterparts.

Our study findings that women’s high educational attainment and high socio-economic status increase women’s odds of ever using HEC, do not come as a surprise. Women’s education might increase HEC use by increasing awareness about its existence and usefulness. In addition, educated women might have higher economic resources to obtain HEC. This is especially important since HEC is mainly obtained by Nicaraguan women through private providers [23]. With an average cost of 2–3 dollars [23] per full dose, HEC might be prohibitive to women of lower socioeconomic status, considering that in 2005, 48% of the Nicaraguan population was below the national poverty line [28]. Our findings are consistent with other population-base studies conducted in Nigeria, Kenia and the US that reported a positive association between HEC use and women’s education [17,18], and with studies conducted among educated populations that have found higher HEC use [19,20,29] when compared with studies conducted among a general population [16,18]. We also found that women’s age was another important factor influencing HEC use in the Nicaraguan setting, with HEC use decreasing with age. These findings are in line with the results of studies conducted in Africa and in the US [17,18]. One possible explanation is that young women might have less access to modern contraceptive methods than older women [23], which increases their risk of engaging in unprotected sex. Hormonal emergency contraception might be one way young women have to avoid pregnancies, as it has been found that HEC use is higher among women having unprotected sex [21].

After adjusting for age, education and socioeconomic status women’s urban residency was found to be positively associated with higher odds of HEC use. Urban/rural differences in HEC use could be explained by differences in access to this post coital contraceptive. As we did not study access to HEC, we could only hypothesize that rural women’s opportunities to obtain hormonal HEC might be limited because their main source of contraceptives are public health care facilities that do not routinely provide HEC [23]. However, further studies are needed to confirm this hypothesis.

### Hormonal emergency contraception and current reversible contraceptive method

Ever use of HEC varied according to the type of reversible contraceptive method currently used. Women using condoms, withdrawal, and calendar-rhythm method reported higher HEC use than those using pills and IUDs. These higher rates might be influenced by the perceived effectiveness of the reversible contraceptive method chosen, or whether the method was not used as recommended. For example, a US cohort study with young women found that inconsistent condom use was a crucial factor influencing the decision to frequently seek HEC [30].

### Hormonal emergency contraception and IPV

Previous studies around the world have shown that women who experience IPV are often exposed to reproductive coercion by their partners [6,7], which often ends in unintended pregnancies [31,32]. However, there is evidence pointing that women are active subjects in trying to counteract their partners’ reproductive coercion. For example, increased reversible contraceptive use has been associated with exposure to IPV in Nicaragua [33] and elsewhere [34]. Our findings show that lifetime exposure different forms of IPV (emotional, physical, sexual IPV, and controlling behavior by partner) are associated with higher odds of ever using HEC even after adjusting for socio-demographic factors. This is a crucial and novel finding that has not been reported before in the Latin American setting. For women exposed to IPV, a pregnancy can hinder their abilities to end an abusive relationship. It might increase their vulnerability by increasing their economic dependency to their partners, and it might weaken women’s ability to assertively respond to their partner’s aggression in order to protect the fetus [35]. Thus, HEC use in this population might reflect women’s strategies to diminish their vulnerability while in an abusive relationship by avoiding an unintended pregnancy. This is especially important in a reproductive context where unintended pregnancies are high [31] and access to therapeutic abortion is banned [25].

### Limitations and strengths

Our study uses cross-sectional data, thus it is not possible to establish a causal relationship between the exposures studied and the use of HEC. Measuring IPV is challenging, especially when questions assessing it are included within a larger survey addressing other topics. It is possible that an information bias in this study underestimates lifetime IPV prevalence, as it is lower than what other studies have found in this setting [33]. Our study sample includes only ever partnered non-sterilized women. This might underestimate HEC use in the general population as it does not include ever use of HEC among sterilized or never partnered women. Our study found significant associations between current socio-demographic variables and ever use of HEC. This might be a limitation since because the woman could have used HEC in the past, when she might had a different age, residence and socioeconomic status.

This study is one of the few nationwide-population based studies around the world that assess inequalities in HEC use among women using a multivariate analysis. This is clearly an advantage since most studies around the world describing HEC use have used clinical-based populations or have been limited to specific populations (i.e. college students), that limit the generalization of their findings. Our study is the first population-base study to have found a direct association between exposure to IPV and increased HEC use. A similar association was found by a recent study conducted among US women, but it was focused on women attending family planning services [36]; which limits it’s results generalizability.

## Conclusion

This study provides sound evidence supporting the hypothesis that there are inequalities in HEC use even in countries where inequalities in use of other forms of contraceptive technology has been reduced. Also, we have highlighted the link between lifetime IPV exposure and ever use of HEC use, which is a key element that can support interventions aiming to decrease unintended pregnancies among women exposed to male partner violence. HEC use among Nicaraguan women is strongly influenced by individual factors such as age, residency, educational level, socioeconomic status, and exposure to different forms of IPV. It is paramount that actions are taken to diminish these gaps.

## Recommendations

HEC is not a daily contraceptive method; however, it must be available as a post-coital contraceptive for those women attempting to prevent an unwanted pregnancy regardless of differences in their socio-economic characteristics and exposure to IPV. Improving its availability might contribute to a decrease in unwanted pregnancies and unsafe abortions in Nicaragua, where abortion is prohibited. In evaluating women’s needs of HEC, health staff must take into account their clients’ reproductive needs, their socio-demographic characteristics, and exposure to IPV.
